# Conversations upon the Physical and Mental Hygiene of Girlhood

**Published:** 1881-03-20

**Authors:** T. S. Powell

**Affiliations:** Atlanta; Professor of Obstetrics and Diseases of Women, and Lecturer on Medical Ethics in the Southern Medical College


### Southern Medical Record.

EDITORS:

T. S. POWELL, M.D. W. T. GOLDSMITH, M.D. R. C. WORD, M.D.

*R. C. WORD, M.D., Managing Editor.*

☞All Communications and Letters on Business connected with the RECORD must be addressed to the Managing Editor.

VOL. XI. ATLANTA, GA., MARCH 20, 1881. NO. 3.

### ORIGINAL AND SELECTED ARTICLES.

### CONVERSATIONS UPON THE PHYSICAL AND MENTAL HYGIENE OF GIRLHOOD.

BY T. S. POWELL, M. D.,

Professor of Obstetrics and Diseases of Women, and Lecturer on Medical Ethics in the Southern Medical College.

### CONVERSATION III.

Mother—"Doctor, good morning. I am glad to see you. Mary is still improving, under your treatment, but I have, since I saw you last, noticed a symptom about her health that gave me a little anxiety."

Doctor—"What is that, madam? Nothing serious, I hope."

Mother—"No, I trust not; but she frequently, after breakfast and supper, complains of palpitation of the heart—at least its pulsation seems quicker than usual, and not altogether regular."

Doctor—"What does your daughter usually eat and drink at breakfast and supper?"

Mother—"Well, usually, bread and butter, eggs, grits, and some kind of sweetmeats, and drinks coffee at both meals. She is very fond of coffee, and likes it rather hot and strong."

Doctor—"The coffee is, no doubt, the cause of the palpitation of her heart. I have observed your daughter is of an excitable temperament, and the ill health she has had for some time has, of course, weakened her nervous system greatly. She ought not to drink coffee, unless it is half milk, and then only at breakfast."

Mother—"All our family are very fond of tea and coffee. Do you think neither of them is a wholesome beverage?"

Doctor—"Well madam, that greatly depends upon the condition, temperament and mode of life of the parties who use it as a beverage; and if tea, whether it is black or green. Both have narcotic and astringent principles. The green tea contains more than the black, and affects more powerfully the nervous system; also, in so many ways, that it is rather an unpopular and unprofitable subject to discuss. For instance, there are some persons who are exhilerated and refreshed by a cup of good tea, either green or black, but it will affect others in an opposite manner, producing great depression, with a variety of nervous symptoms. There is still another class of persons who are not benefitted by the use of tea—I mean those who suffer from indigestion in any form. Hence, there is no beverage of which a greater variety of opinions have been expressed."

Mother.—"Yes, I have heard various opinions advanced about tea as a drink, but none of them seemed satisfactory."

Doctor—"Some writers regard tea as a poison; others recommend it as a valuable stimulant, and go so far as to say that it assists the digestive organs in their office. I think all these opinions are true, if applied with discrimination to the classes of persons I have mentioned. If taken in moderate quantity, at a moderate temperature, and at proper times, unpleasant effects are seldom produced.

It is certainly injurious to drink of tea copiously during meals or soon after. Some think that in such quantity it interferes with digestion by diluting the gastric juice, distending the stomach, and retarding chymification on account of its narcotic and astringent principles. It is evident that if the stomach is greatly distended with a fluid of any kind, it will be more difficult for the solids to be digested; but as to the fluid diluting the gastric juice, I am not fully prepared to endorse this opinion, because the gastric juice is not very soluble in water. At the same time it is true that this important element in the process of digestion cannot act upon solid food when too much fluid has been taken into the stomach. On the other hand, if the solid food is of a dry, hard nature, the fluid must be sufficiently copious to soften its solidity, or digestion will be equally if not more impeded. Therefore, different kinds of food require an intelligent discrimination as to the quantity of fluid to be taken while eating, so as to perfect chymification or digestion. Now, madam, a few words on the subject of coffee."

Mother—"Before you give me your opinion upon coffee, please tell me what kind of food requires more or less fluid to promote the proper digestive action, and at what times should it be taken."

Doctor—"I do not know that I can answer these questions conclusively, as hygienists differ upon the subject. Besides, their various opinions would probably be of no especial benefit to you, and it would consume so much time to enumerate them that I fear it would tax your patience."

Mother—"No, indeed, Doctor. I would listen attentively, and try to understand. I am very much interested in the subject."

Doctor—"I think you are, madam, and I see you have one beautiful and profitable accomplishment, not possessed by all of your sex—that of being a charming listener—an accomplishment as much needed in the discussion of these plain but important questions as in the most brilliant topics of the drawing-room. In my preceding remarks I stated that food of a dry or hard consistency required more liquid than vegetables, as most of the latter have but little solidity after being prepared for the table. You know that more or less liquid is also required in cooking different articles of food, as baked meats must have more liquid in preparation than if roasted, and boiled meats must have more than either. As to the best time for taking liquids, I have also partially answered, but will be more explicit by saying that too much liquid drank just before, just after or during meals, will certainly interfere with the stomach in properly performing its natural functions; but if the nature of the food requires it, a small amount of liquid really assists digestion. There is but little food ever eaten that is so dry as to call for more than merely sipping the tea or coffee, or whatever fluid is used. It is gulping down many cups or glasses of the beverage that does the harm."

Mother—"Yes, I know that is true, and as an illustration, I used to think that I could not eat fresh fish, mackerel, etc., because it made me so very thirsty it seems I could not help drinking water until I was threatened with congestion of the bowels, and felt miserably for hours after eating. Some one then told me if I would control my thirst and drink no water at all for three or four hours after eating fish, I would find that it was the copious drinking of water that made me sick, and not the fish. I tried it, and found it true to my great gratification, and not only in regard to fish, but other dishes, and all fresh or salted meats."

Doctor—"Certainly, madam, because, as I have said, food, especially meats, cannot be well or quickly digested when floating in a large quantity of water or other liquid in the stomach. I know a lady who ate moderately of fried fresh fish one day at dinner. During the afternoon she drank, I suppose, more than a quart of cold water, and about 6 o'clock that evening she was taken with congestion of the bowels, and came very near dying. Now, every one in shameful ignorance of the laws of health, would say it was the fish that made her sick, when, in reality, it was the large quantity of water she had drank. The fish was fresh from the river, delightfully cooked, delicious and wholesome in every respect, but it had to bear the responsibility of the attack of almost fatal illness. The proper time to take liquids is between meals, but not in these copious quantities. The only time when it is best to drink anything soon after eating, is when frozen fruits or ice-cream have been eaten at the table, as at dessert, for instance. Then a very small cup of coffee or tea pleasantly hot, can be taken with benefit, because it neutralizes the chilling effects of the iced viands. Digestion cannot be brought about perfectly when the stomach is either chilled by frozen articles of food, or unnaturally heated by those eaten almost scalding hot. Later after eating, water may be drank moderately without any injury, and indeed is then often necessary for the re-supply of our fluids as the chyle is being assimilated, and entering the blood to undergo its last change, and a certain quantity of liquid is now needed to complete its operations, and carry off the unnutritious matter."

Mother—"Do you think tea is more wholesome than coffee?"

Doctor—"Coffee is the more stimulating, but like tea, has dissimilar effects upon the nervous system of different persons. In some instances it prevents sleep, and in dyspeptics frequently produces acidity of the stomach, but with most persons a small cup of coffee taken at meals does not disturb digestion as tea is often found to do, but really increases and quickens the forces of the stomach, and enables some dyspeptics to digest articles of food that without it would have given great distress. When taken in such cases, for this purpose, it is best to have the coffee made by infusion or boiling, so that much of the aroma may be thrown off before drinking. If agreeable to the taste, a little sugar may be used, as this does not change the properties of the coffee, but milk weakens the effects and should not be used when the drink is taken as a promoter of digestion."

Mother—"But if the milk lessens the stimulating effects of the coffee, it is better for nervous people to use it in that way, is it not?"

Doctor—"Decidedly, and if they have a distaste for the milk, and the coffee is a strong infusion, it should be diluted with hot water or what is better, beat the yolk of a fresh egg in the cup, add the sugar, then stir in the coffee. A cup of tea or coffee prepared in the latter manner is very nourishing, especially suited to invalids, and all nervour persons who use coffee or tea, as the addition of the egg seems to lessen the stimulating properties of these beverages. It also gives it a rich and pleasant flavor, and is preferable to cream or milk."

Mother—"If tea nor coffee is good for our children, I am satisfied they have been a great injury to many. Would it be better to give them chocolate or cocoa?"

Doctor—"I know but little of them experimentally, as I have never been fashionable enough to use either; but in my judgment both are injurious as a beverage. They contain so much nutritive matter, they should be considered more as a food than a drink. Chocolate has a much larger amount than cocoa, and is also adulterated with various articles which make it more liable to disagree with the stomach, and often produces more or less nervous excitation. It also contains an oil which is so difficult to assimilate, that it furnishes but little nourishment to the system, and that little at great expense to the functions of the stomach. Cocoa contains less oil, and less nutrition, and is therefore not so objectionable as a beverage, nor so apt to interfere with digestion, but neither should be used by children or invalids. But madam, I must go now. Mrs. ———, who has been expecting me for two hours, will give me—well, you know how she can bless a fellow."

Mother—"Never mind about Mrs. ———, she is a good woman if she does sometimes get into a pet, and say ugly words. If tea, chocolate nor coffee is good for children, do tell me what should I give mine to drink."

Doctor—"There is one kind of tea that is good for them, madam, and is also really delicious when properly prepared. It is an old fashioned drink, and what our mothers and grand-mothers called 'kettle tea.' It is made by putting the teacup one-third full of fresh, rich sweet milk, then poor in water *boiling* hot until the cup is full, add as much white sugar as is desired, and if the milk is *pure* as well as fresh and sweet, you now have a wholesome drink, and one that all children will think delicious."

Mother—"Oh, yes, I have often prepared that kind of tea for my babies, but thought it only suited to children *as* babies."

Doctor—"It is suitable for them at any age, my dear madam, even after they become men and women, and I have known not only rosey, cheeked girls, but great rollicking boys, who had been accustomed to this drink all their lives, to actually cry for it if it was missing at the time. They usually took it with their food. These careful and intelligent mothers had never allowed their tender stomachs to be disordered by coffee and green and black tea, and their nervous system to become morbidly excited by the stimulating effects of either, consequently their taste for drinks was not perverted and manifested by a craving for artificial stimulants, but they only desired their natural and wholesome beverage—pure milk and water. I am satisfied that table teas and coffee are highly stimulating drinks, and are not only injurious to children's health at any age, but also causes them to be restless in their sleep, and more cross and excitable when awake than they would be under other circumstances."

Mother—"Then you advise me to give my children the 'kettle tea."

Doctor—"Yes, madam, you and all mothers. In the winter season, give them 'kettle tea' at breakfast and supper, and sweet milk alone at dinner. During the warm months let them also have 'kettle tea' at breakfast, and fresh butter milk at dinner and supper. If the milk cannot be procured I would advise all mothers to give their children only cold water."

Mother—"Since you have mentioned this, Doctor, I remember how I have been surprised and grieved to see my children often so nervous and irritable, when otherwise they seemed to have very amiable dispositions. I shall certainly make this change in their diet at once, but as they have used a great deal of coffee and both black and green teas, I expect they will rebel at being deprived of the stimulants."

Doctor—"They may for awhile, but persevere in it; tell your children kindly but firmly, why such drinks are not good for them, and I am certain they will soon become fond of the kettle-tea. I have frequently observed that when children were physically in a normal healthful condition, their instincts in the choice of the most wholesome food was greater than is shown by grown up people."

Mother—"I have often noticed that in children myself, and wondered why it was so. I now see that it is instinct. I see, Doctor, you are getting impatient and anxious to go, but I have a few more questions to ask you, which I know you will answer. Do tell me what you think of wines and all spirituous liquors as a beverage, and as a medicine or tonic?"

Doctor—"I do not think that the moderate use of pure wines and liquors, taken at the proper time, is positively injurious to adults. Sometimes a small quantity of pure wine, brandy or whisky, taken daily by persons advanced in age, say sixty years, is of real benefit in gently toning up the waning powers and strength consequent upon that stage of existence. Even then, it is not really necessary if old people are in perfect health, and have been accustomed to an active out-door life. The quantity of teas and coffee that most old people indulge in is generally sufficient without any drink more stimulating. To younger persons and infants in perfect health, spirituous liquors are certainly an injury in every instance. Where they are really needed by persons in ill-health, I consider it a medicine, not as a beverage. But in some cases where it is used as a medicine, it is not needed as such if those who use it would change their mode of living. This is true of many persons who are considered healthy, who have no positive disease as yet, but there is some little disorder, some diversion from perfect health, caused by living up to the requirements of society, and they now feel it is necessary to use a little stimulant to keep up the usual strength to meet the demands of a life that is continually exhausting the physical powers. But like all artificial strength, it is only temporary, and the person is more feeble than before, and more depressed in spirits as soon as the stimulant is withdrawn."

Mother—"Yes, I know that is always the result when liquors are used as a stimulant by ladies of society. But are they not good as dyspeptic tonics?"

Doctor—"They are of some benefit in a few instances of atonic dyspepsia, but the good that they do in that form of dyspepsia has been much exaggerated, and has caused them to be used in irritable and inflammatory cases, and which greatly aggravated the symptoms. So, where one person in ill-health has been benefitted by the stimulants, others have been damaged."

Mother—"Do all pure wines contain alcohol?"

Doctor—"Yes, the stimulating and intoxicating element of all spirituous liquors is alcohol. Some contain more than others, and affect the system according to its combinations and conditions. For instance, the same amount of alcohol taken in the form of pure wine or greatly diluted with water—especially if it has been mixed for some hours before drinking—affects the system very differently, as regards intoxication, than if it was drank in the form of whisky or brandy. This is because alcohol contained in wine is more readily blended with water than when it is mixed with other ingredients, and therefore, it does not intoxicate as it would if drank without this preparation. This proves the truth of every man's experience, that the same wine affects the same man differently at different times, according to the state of the stomach when the wine is taken."

Mother—"Do you think, Doctor, that beer and ale are unwholesome, as beverages?"

Doctor—"Well, madam, it can be said of all malt liquors, as others, that sometimes they are of a little benefit to the person who uses them in moderation, while they have proved of great injury to others. They are unlike wines in many respects: first, they contain a peculiar bitter and narcotic property which they receive from the hops used, in their manufacture; they contain less spirituous and more nutritive matter, and we find that those persons who use these drinks freely are generally fat, and in many cases when these are immoderately taken with a strong course of diet, if the persons do not habitually exercise to a great extent, they soon find themselves in a plethoric condition, the secretions, to a great extent, arrested and vitiated, thus producing disorders and diseases. This explains why malt liquors are not as injurious to the health of the poor and the laboring classes as to the rich, because the latter take less exercise in doors and out, and at the same time live luxuriously. The amount of nutritive matter contained in beer or ale, is not very easily digested, and when taken in connection with the variety of rich food commonly found on the table of the wealthy classes, serious damage to health is almost sure to eventually follow."

Mother—"I see one must use discrimination upon these points to determine when these drinks are injurious."

Doctor—"Yes, madam, one must understand and observe these points, and many others in regard to the subject, or a large majority of the sick, and those who are not, yet habitually use malt liquors, will sooner or later become diseased, and fatally so in many cases. Ah, good morning, Miss Mary; I am glad you have come in before I left. You are still improving, I hope."

Patient—"I believe I am almost well. I can work, eat and sleep so much better."

Doctor—"Yes, it is very evident that health is returning, and much faster than I at first anticipated; and, my dear madam, I must say that it is owing, in a great degree, to your intelligent watchfulness in enforcing obedience to my prescriptions and the proper hygienic measures for the restoration of your daughter's health. I thank you for your co-operation, and if all parents would thus assist the attending physician, "the Doctors" would not so often be charged with failure in having done their whole duty."

Mother—"Yes, Doctor, that is true, for, while I know there are quacks among many so-called "Doctors," I am sure that persons very often, even when they have the best of physicians, expect more of him than he can really perform. I know "the Doctor" cannot cure any one who is sick, unless the patient has intelligent and careful nursing and follows directions implicitly."

Doctor—"And, yet, madam, we have so few really good nurses—those who are conscientiously faithful, and have a full and intelligent knowledge of the important position."

Mother—"Yes, very few, indeed. I sometimes think that no woman or young girl ought to be considered accomplished unless she has had a good training in this department."

Doctor—"Her home accomplishments are certainly deficient without this very desirable one; and if you can get the ladies to look upon the proper nursing of a sick person as an accomplishment, they will probably devote a part of the time to its acquisition, that they now spend in weeping over the sham sorrows of heroines in dime novels, or on the stage. But, Miss Mary, though you have greatly improved in every respect, yet you are not quite well, and I want you to get ready and go to your uncle's in the country. The summer months will begin in a few days."

Patient—"Well, Doctor, I can go at any time, will start to-morrow, if you say so."

Doctor—"Very well, pack your trunk at once. I want to get you where you can romp and play under the trees, eat good, fresh food, and drink pure spring water."

Mother—"I am glad you mentioned water, Doctor. I have been wishing to ask your opinions about the different kinds. We have lived where we were compelled to use a variety of waters, spring, well, rain, river, lake and even marsh water."

Doctor—"Water is certainly God's beverage for man, but its quality and healthfulness depends upon the source from which it is derived. Water that contains a large amount of earthy matter is without doubt a great source of certain diseases. It is still more unwholesome when it has in solution putrescent animal or vegetable matter, or is strongly impregnated with certain minerals. It is for the first-mentioned reason that some water is wholesome one year and the next is the cause of disease."

Mother—"Mary, you can go now, if you wish, as you do not seem to be interested in our conversation."

Doctor—"Yes, madam, I think your daughter takes an interest in our talk, at least she ought to be interested more, perhaps, than yourself. The popular idea that mothers should not have their daughters learn from the proper sources everything that is necessary to preserve their health in a perfect condition, has destroyed many promising young lives, and made desolate many a mother's heart. All these things can be taught by the mother in an earnest, impressive, and yet confidential and delicate manner, so as not to offend the modesty of the most chastely-nurtured young girl, nor intimidate her confidence in seeking and obeying her mother's counsels whenever it is necessary."

Mother—"Yes, Doctor, I know I had fallen into that popular idea to a great extent myself, and as I now feel, much to my regret, for since my talk with you on these subjects, I can see more fully how remiss I have been in this important duty."

Doctor—"But I am sure, my dear madam, that you will not be so in the future. Conviction is the first step towards action. But, let us return to the subject of water. Rain water, if caught in the open field, is the purest of all water, but when caught in large pits it is impregnated with impurities from the smoky and impure atmosphere through which it passes, and very often becomes contaminated with calcareous matter which makes it necessary to boil and strain the water before it is fit for use. On the whole, spring water is perhaps the purest for domestic or medicinal purposes, especially when the springs are large and run out of silicious rocks or beds of gravel—in either case the water is less impregnated with impurities, and the minerals that are not conducive to health. This is pure soft water. Even horses often become diseased from being compelled to drink hard water all the time. As pure water is so essential to health and long life, no expense should be spared to obtain it. When you first procure it, well water is the same as that from a spring, either soft or hard, according to the subsoil or geological formations through which it passes; but well water is more likely to become impure from infiltration, as the least trace of filth from a cess-pool or drain may, and often does, convert well water into a slow poison, producing disease and death."

Mother—"Doctor, how are we to determine that the water is thus contaminated? Does it affect the color or taste?"

Doctor—"No, madam, very often water thus polluted is pleasant to the taste, it is clear and sparkling, and no danger is suspected until some serious disease breaks out in the family, and of a nature to create suspicion that filth is the cause. The recent investigations into the causes of cholera has confirmed the opinion that the drinking of impure water is, to a great extent, the basis of this dreaded and fatal disease. The diseases that are caused by the use of impure water are typhus fever, diphtheria, diarrhœa and dysentery. The only certain test of impure water is to submit it to chemical analysis."

Mother—"But, Doctor, why is it said that the water of old wells is pleasanter and purer than that of new?"

Doctor—"Because the unpleasant and unhealthy particles of matter found in nearly all new wells have been drawn off, and for the same reason, when a well or spring has a bold vein flowing all the time, the more water is used the better it becomes."

Mother—"Yes, I was aware of that, but I never knew the philosophy of it before."

Mother—"But, Doctor, how can we get pure drinking water in a city, especially, when we have such a miserable, inefficient system."

Doctor—"The next, and I believe the last water upon which you desire my opinion is the lake or marsh water. Both are entirely unfit for man or beast. But madam, my time is now out, and I must ask to be allowed to tell you the reason why this water is poisonous at my next visit, I must go, good morning. I will call this day week."

Mother—"I shall look for you. You must not disappoint me."

Doctor—"No madam, I will not. You may look for me with certainty, unless some unforeseen circumstance should intervene so as to prevent me. Punctuality in visiting patients, and fidelity in complying with engagements, I teach my students is very essential to inspire confidence on the part of the patient in his physician. Good morning."

[TO BE CONTINUED.]

